# Clinical and epidemiological characterization of influenza virus infections in children with severe acute respiratory infection in Maputo, Mozambique: Results from the implementation of sentinel surveillance, 2014 – 2016

**DOI:** 10.1371/journal.pone.0194138

**Published:** 2018-03-28

**Authors:** Neuza Nguenha, Almiro Tivane, Mirela Pale, Loira Machalele, Afonso Nacoto, Germano Pires, Edirsse Mationane, Judite Salência, Félix Gundane, Délcio Muteto, Josina Chilundo, Sandra Mavale, Noorbebi Adamo, Cynthia Semá-Baltazar, Orvalho Augusto, Eduardo Gudo, Tufária Mussá

**Affiliations:** 1 National Institute of Health, Ministry of Health, Maputo, Mozambique; 2 Pediatric Departament, Hospital Geral de Mavalane, Maputo, Mozambique; 3 Pediatric Departament, Hospital Central de Maputo, Maputo, Mozambique; 4 Faculty of Medicine, Eduardo Mondlane University, Maputo, Mozambique; Lecturer, Discipline of Paediatrics, School of Women's and Children's Health, UNSW Medicine and Research Scientist Sydney Children’s Hospital, AUSTRALIA

## Abstract

In Sub-Saharan Africa, where burden, impact, and incidence of acute respiratory infections (ARI) are the highest in the world, conversely, the epidemiology of influenza-associated severe acute respiratory infections (SARI) is incompletely known. The aim of this study was to describe the clinical and epidemiological features of influenza-associated SARI in hospitalized children in Maputo city, Mozambique. Nasopharyngeal and oropharyngeal swabs were collected from children aged 0–14 years old who met the case definition for SARI in two hospitals in Maputo city after their parents or legal representative consented to participate. A structured questionnaire was used to collect clinical and demographic data. Typing and subtyping of influenza were performed by real-time PCR. From January 2014 to December 2016, a total of 2,007 eligible children were recruited, of whom 1,997 (99.5%) were screened for influenza by real-time PCR. The median age of participants was 16.9 months (IQR: 7.0–38.9 months) and 53.9% (1076/1991) were male. A total of 77 were positive for influenza, yielding a frequency of 3.9% (77/1,991), with the highest frequency being reported in the age group 1–5 years old. Cases of influenza peaked twice each year, during which, its frequency reached up to 60%-80%. Among all influenza confirmed cases, 33.7% (26/77), 35.1% (27/77) and 28.6% (22/77) were typed as influenza A/H3N2, A/H1N1pdm09, and B, respectively. This represents the first report of influenza in urban/sub urban setting in Mozambique and the first evidence of distribution of strains of influenza in the country. Our data showed that frequency of influenza was lower than reported in a rural setting in Mozambique and the frequency of seasonal (A/H1N1pdm09) and (A/H3N2) subtypes were similar in children with SARI.

## Introduction

The World Health Organization (WHO) estimates that worldwide, 20–30% of children are infected with the influenza virus each year, causing 1 to 2 million cases of Severe Acute Respiratory Illness (SARI) and up to 100,000 progress to death each year. Of these, up to 90% are known to occur in developing countries [1]. Sub-Saharan Africa still remains one of the most affected regions, accounting for almost 50% of cases of ARI and 20% of cases of cases of influenza-associated SARI in children worldwide [2–7]

Although now we know more about influenza in sub-Saharan Africa compared to 10 years ago [8–10], available data is still insufficient to inform the development of preventive and control strategies in the continent and gaps in terms of representativeness still exist. In Sub-Saharan Africa, the high burden of comorbidity conditions, including HIV, tuberculosis, and under nutrition, combined with the limited access to health care services may lead to worse outcomes associated with influenza infection [11].

Although several studies conducted in the Sub-Saharan Africa suggests that the burden of influenza is significant [12–14], the disease remains heavily neglected in many countries in the region, including Mozambique, where no surveillance system for influenza exists.

On the other hand, the seasonality of influenza virus is heterogenic in different regions worldwide, for instance, while in most of the countries in the tropics, influenza occurs, both in the dry and rainy season [15–18], in the temperate climate, influenza occurs mostly in winter season [19]. In this context, it's important to understand the seasonality of influenza in each country as country-to-country variation also occur.

Lack of local information on the epidemiology of influenza-associated SARI represents an important barrier to the definition and implementation of interventions to reduce its burdens, such as vaccination and anti-viral treatment targeting high-risk groups.

Another important aspect to consider is that in Mozambique, *Haemophilus influenzae type b* and *Streptococcus pneumoniae* vaccination was introduced in the immunization schedule in 2009 and 2013, respectively, which may have led to a reduction in the burden of bacterial pneumonia. In this context, there is an increasing concern that respiratory virus, including influenza viruses, may have become a leading cause of pneumonia in children, but data on the epidemiology of influenza-associated SARI in children are scarce. In Mozambique, Influenza vaccine is not available in the public sector and no approved guidelines exist for influenza vaccination. No vaccine had been purchased in the public sector in the country. Similarly, no antiviral is available in the public sector in Mozambique. In regard to private sector, no control exists on the use of influenza vaccines or antivirals. The few studies conducted so far were all from a small rural village in southern Mozambique[20–22], which limits extrapolation to other areas of the country, particularly in a time in which the country is experiencing a rapid growth of people living in highly crowded sub urban settings [23]. On the other hand, no study had been conducted to assess the distribution of strains of influenza virus in Mozambique. Thus, the aim of this study was to describe the clinical and epidemiological features of influenza-associated SARI in hospitalized children in Maputo city, Mozambique from January 2014 to December 2016. Our study is the first to be conducted in hospitalized children from urban/sub urban settings in Mozambique and the first that describes the most common types and sub-types of influenza circulating in children with SARI in Mozambique.

## Material and methods

### Study setting and participants

**Fig 1:**
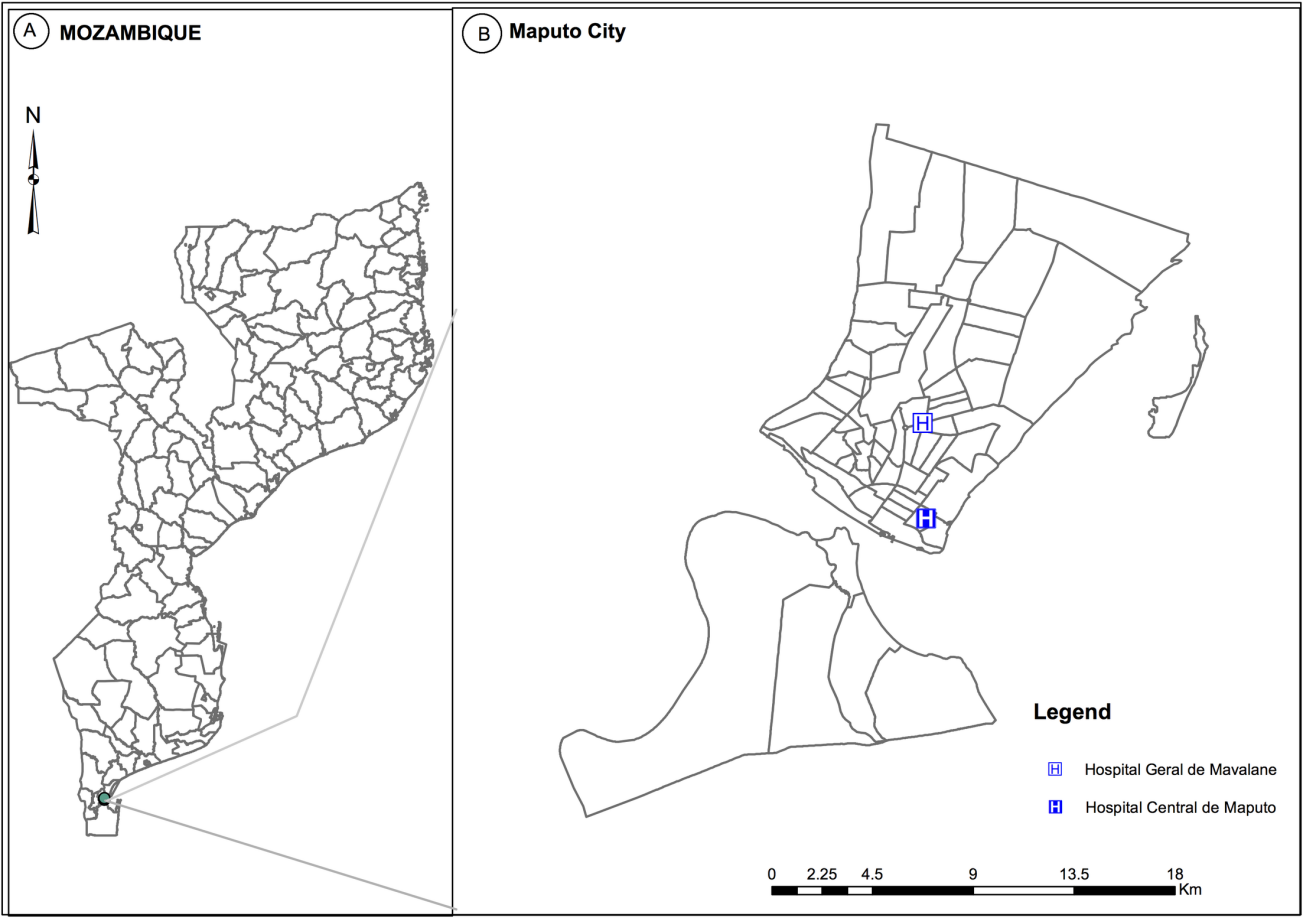
Geographical representation of the study area. The left panel shows the geographical localization of Maputo city in the Mozambique map. The right panel shows the location of Maputo Central Hospital (H in the blue box) and Mavalane General Hospital (H in the white box), respectively.

This study was conducted as part of the national sentinel surveillance system for influenza in Mozambique. Influenza surveillance system was established by the National Institute of Health in 2014 at two hospitals in Maputo city (Fig 1A), the Maputo Central Hospital and the Mavalane General Hospital, respectively (Fig 1B). These hospitals were selected as they are the main hospitals in Maputo city, the capital of the country. They serve and are representative of the urban/sub urban population of Maputo city. In addition, both hospitals have a paediatrics ward and paediatrics intensive care. Maputo Central Hospital, with a total of 323 beds in the paediatrics ward, is the largest hospital in the country. Mavalane General Hospital is situated in the sub-urban area of Maputo city and has a total of 68 beds in the paediatrics ward.

At each hospital, inpatient children from 0 to 15 years old, who met the World Health Organization (WHO) case definition for SARI were recruited by a trained physician. Participants were recruited between January 2014 and December 2016. Nasopharyngeal and oropharyngeal swabs and epidemiologic data from SARI cases were collected by trained clinicians or nurses in average 24h after admission.

The climate in Maputo is tropical humid with two distinct seasons, rainy (wet) season from November through April and the dry season from May to October. The total population of Maputo city is 1,257.453 inhabitants [24].

### Case definition

As per WHO guidelines, a SARI case is defined as a patient acute respiratory infection with self-reported fever or measured fever (>38°C), cough, with onset within the last 10 days and requires hospitalization [25].

A confirmed case of influenza was defined as a patient with a positive result by real-time PCR for influenza.

### Enrollment of participants and data collection

All SARI cases admitted to the pediatric wards at the two hospitals were eligible for enrollment. However, because a large number of children with SARI are daily admitted to both hospitals, each day only the first three recently admitted SARI cases were systematically enrolled in order to minimize selection bias. The number of children recruited daily was defined based on the available capacity for laboratory testing and to ensure a high quality of sample collection and also a high rate of completeness of case investigation forms. On a daily basis, the identification of SARI cases to be enrolled was performed based on the time of admission, which was available in the logbook at paediatrics ward. Consent to participate was requested to each child's parent or legal representative before enrollment.

Upon enrolment, a standardized case investigation form (CIF) (S1 File) was completed for each participant. The form contained information on demographic characteristics (age, sex, weight, height, address), vaccination (BCG, DTP, HepB, PVC10, Measles and Influenza), reason for hospitalization (bronchopneumonia, pneumonia, bronchitis, tuberculosis or suspected tuberculosis, asthma), clinical presentation (fever, cough, chills, difficulty breathing, sore throat) co-morbidities (asthma, diabetes, chronic liver disease, cardiovascular disease, neuromuscular disease) and HIV status. Data were collected by the clinicians or nurse, either by reviewing the patient file (demographic characteristics, clinical history, and presentation, HIV status) or and by interviewing the child’s legal representative or caregiver (risk factors for severe disease, previous symptoms, duration of symptoms, antibiotic treatments prior hospitalization.

### Specimen collection and laboratory testing

Two flocked plastic/polyester swabs (Becton Dickinson, USA, MD) were used to collect a nasopharyngeal and an oropharyngeal sample from each patient. Then the swabs were placed in a vial containing 3 mL of virus transport medium (VTM) with antibiotics and sent on the day of their collection, at 2−8 °C, to the Virus Isolation Laboratory (LIV) which is located on within the campus of the Maputo Central Hospital, and 20 minutes away from the Mavalane General Hospital if driving. At the LIV, swabs were removed and the VTM from each sample was split into two aliquots and stored at −70 °C. One hundred and forty micro litres of samples were used to extract RNA using QIAamp viral RNA mini kit (Qiagen Inc., Valencia, Spain), following the manufacturer instructions. One-step RT–PCR was carried out using the Human Influenza Virus Real-Time RT-PCR Diagnostic Panel developed by the Center for Diseases Control and Prevention (CDC), Influenza Division (USA, Atlanta). Specimens with a crossover threshold (*C*_T_) values ≤ 38 were considered positive. Specimens found positive for influenza A virus were subsequently sub typed for seasonal H1 and H3, using another real-time RT–PCR with primers, probes and positive controls provided by CDC-Atlanta (Quiagen, USA, Atlanta).

### Data analysis

Descriptive statistics, including calculation of frequencies of categorical variables. Per demographic and clinical characteristic, the proportion of influenza positive tests were computed. Chi-squared test and Mann-Whitney test were used to compare influenza positive and negative children in terms of their demographic and clinical characteristics. For proportions, we report the binomial exact 95% confidence-intervals. To estimate time trends of the proportion of influenza positive tests per each demographic and clinical characteristic we fit a log-binomial regression with calendar time, the dummy of the levels of the characteristic and the calendar time and dummies interaction terms. Only the overall p-value of the interaction is reported to test for heterogeneity of time trends per characteristics. All analysis were performed using Stata software package (College Station, Texas: StataCorp, USA, 2005). The significance level was set at 5%.

### Ethics statement and consent to participate

The study was approved by the Mozambican National Bioethics Committee (Ref #: IRB00002657). Verbal consent was obtained from the legal representative of each child as per the requirements of the routine sentinel surveillance system.

## Results

### Characteristics of SARI children

**Fig 2:**
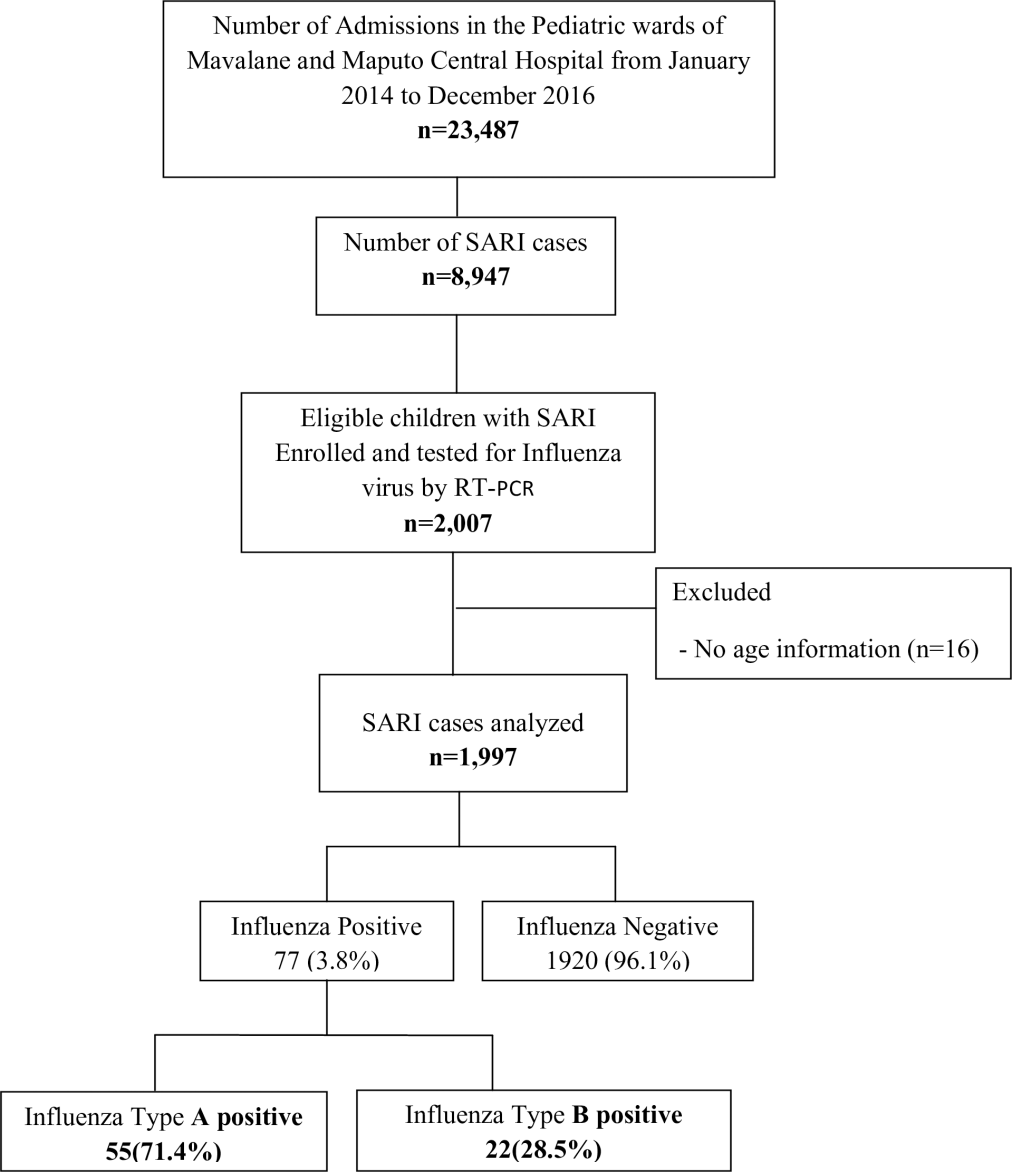
Flowchart of patient recruitment and sample testing. From January 2014 through December 2016, a total of 23.487 children were seen at the Pediatric ward of Mavalane General Hospital and Maputo Central Hospital, of whom 8,947 were admitted with SARI. Of these, 2,007 patients were enrolled and screened for influenza virus, of whom, a total of 16 were excluded due to lack of information on their age, yielding a final sample of 1,997.

From January 2014 to December 2016, a total of 2,007 eligible children were recruited at two sentinel hospitals in Maputo and tested for influenza using RT-PCR, of whom 16 were excluded from the study because of lack of information on their age (Fig 2).

In this context, the final sample comprised a total of 1,997 children, corresponding to 22.3% (1,997/8,947) of the total number of children hospitalized with SARI during the same period.

The median age of SARI children was 16.9 months (IQR: 7.0–38.9 months) and 1076 (53.9%) were male (Table 1).

**Table 1:** Clinical and demographic characteristics of study participants.

	SARI	Flu Negative	Flu Positive	Influenza-positive	p-value
Characteristic	N (%)	N	N	% (95%CI)	
Total	1997 (100.0)	1920 (100.0)	77 (100.0)	3.9 (3.1–4.8)	
Age in months*					
Min–Max	0.1M - 14.0Yr	0.1M - 14.0Yr	1.0M - 14.0Yr		
Median (IQR)	16.9 (7.0–38.9)	16.8 (7.0–38.6)	18.8 (9.8–44.3)		0.170
**Categories**					0.194
< 6	435 (21.8)	426 (22.2)	9 (11.7)	2.1 (1.0–3.9)	
6–11	331 (16.6)	317 (16.5)	14 (18.2)	4.2 (2.3–7.0)	
12–23	444 (22.2)	423 (22.0)	21 (27.3)	4.7 (3.0–7.1)	
24–59	493 (24.7)	474 (24.7)	19 (24.7)	3.9 (2.3–6.0)	
5Yr– 14Yr	294 (14.7)	280 (14.6)	14 (18.2)	4.8 (2.6–7.9)	
**Gender**					0.633
Male	1076 (53.9)	1032 (53.8)	44 (57.1)	3.6 (2.4–5.0)	
Female	842 (42.2)	812 (42.3)	30 (39.0)	4.1 (3.0–5.5)	
No information	79 (4.0)	76 (4.0)	3 (3.9)	3.8 (0.8–10.7)	
**Season of case detection**					0.154
Wet	793 (39.7)	756 (39.4)	37 (48.1)	4.7 (3.3–6.4)	
Dry	1204 (60.3)	1164 (60.6)	40 (51.9)	3.3 (2.4–4.5)	
**Signs at hospitalization**					
Self-reported fever	900 (45.1)	855 (44.5)	45 (58.4)	5.0 (3.7–6.6)	**0.019**
Difficult breathing	1150 (57.6)	1106 (57.6)	44 (57.1)	3.8 (2.8–5.1)	1.000
Measured fever (> 38C)	75 (3.8)	70 (3.6)	5 (6.5)	6.7 (2.2–14.9)	0.209
**Symptoms at hospitalization**					
Cough	1808 (90.5)	1732 (90.2)	76 (98.7)	4.2 (3.3–5.2)	**0.008**
Sore throat	101 (5.1)	97 (5.1)	4 (5.2)	4.0 (1.1–9.8)	0.794
Runny nose	1104 (55.3)	1057 (55.1)	47 (61.0)	4.3 (3.1–5.6)	0.350
Reason for hospitalization**					
Bronchopneumonia	1207 (60.4)	1160 (60.4)	47 (61.0)	3.9 (2.9–5.1)	1.000
Pneumonia	251 (12.6)	247 (12.9)	4 (5.2)	1.6 (0.4–4.0)	0.052
Bronchitis	259 (13.0)	255 (13.3)	4 (5.2)	1.5 (0.4–3.9)	**0.037**
Other (non-respiratory)***	383 (19.2)	360 (18.8)	23 (29.9)	6.0 (3.8–8.9)	**0.025**
Previous or currently diagnosed asthma	558 (27.9)	535 (27.9)	23 (29.9)	4.1 (2.6–6.1)	0.699
**Treatments**					
Antibiotics	1444 (72.3)	1387 (72.2)	57 (74.0)	3.9 (3.0–5.1)	0.796
Oxygenation	239 (12.0)	227 (11.8)	12 (15.6)	5.0 (2.6–8.6)	0.287
Other (no oxygenation neither antibiotics)****	526 (26.3)	509 (26.5)	17 (22.1)	3.2 (1.9–5.1)	0.431
**Outcome**					0.179
Death	5 (0.3)	4 (0.2)	1 (1.3)	20.0 (0.5–71.6)	
Recovered	1992 (99.7)	1916 (99.8)	76 (98.7)	3.8 (3.0–4.8)	

*M—months; Yr–years.

** Some cases had multiple diagnoses.

*** Malaria, Oral Candidiasis, Anemia, Acute Gastroenteritis, Marasmus, Kwashiorkor, Malnutrition and Congenital Cardiopathy.

**** Mechanical ventilation and admission to the Intensive Care Unit.

The most frequent symptom in children with SARI was a cough (90.5%; 1808/1997), which was significantly more frequent in influenza-positive children as compared influenza negative children (p = 0.008).

Difficulty in breathing was the most frequent sign in children with SARI (57.6%; 1150/1997) and frequency of self-reported fever was significantly higher in influenza-positive children (p = 0.019).

Bronchopneumonia was the main reason for admission among children with SARI (60.4%; 1207/1997) and Bronchitis was significantly less frequent in influenza-positive children (p = 0.025). Other non-respiratory diseases were more frequent in influenza-positive children (p = 0.037). Asthma was a frequent comorbidity and was reported in 27.9% (558/1997) of children with SARI.

A total of five deaths were reported among children younger than 5 years with SARI, and all of them had been admitted with the clinical diagnosis of bronchopneumonia. The median duration of hospitalization was 5 days.

No patient had history or record of the use of influenza vaccine (see S2 File).

### Characteristics of influenza positive patients

Of the 77 SARI children with laboratory-confirmed influenza, 23 (29.9%) were aged ≤1 years old, 40 (52.0%) were children aged between 1 and 5 years old and 14 (18.2%) were children aged between 5 and 14 years old (Table 1). The median age of SARI children with laboratory-confirmed influenza was 18.8 months (IQR: 9.8–44.3 months) and 44 (57.1%) were male.

The frequency of influenza positive cases was slightly higher in the dry season as compared to wet season (51.9% vs 48.1%, *p* = 0,154).

Patients with confirmed influenza had a significantly higher frequency of a cough (98.7% vs 90.2%, p = 0.008) and self-reported fever (58.4% vs. 44.5%, p = 0.019) as compared to influenza negative SARI cases. The frequency of a runny nose and measured fever was slightly higher in patients with confirmed influenza, but this difference did not reach statistical significance. Patients with confirmed influenza had a significantly lower frequency of bronchitis (5.2% vs 13.3%, p = 0.037) There was the trend towards a lower frequency of pneumonia in patients with confirmed influenza (5.2% vs 12.9%, p = 0.052).

The frequency of asthma in influenza-positive participants was similar to that reported in influenza negative participants (0.699).

The majority of SARI children with and without confirmed influenza were treated antibiotic (74.0% in influenza confirmed children vs. 72.2% in influenza negative children, p = 0.796).

There was a heterogeneity in the number of SARI children enrolled in different years, as the numbers of enrolled children in 2014, 2015 and 2016 were 168, 1133 and 697, respectively, which shows that the largest number of SARI children were recruited in 2015 and the lowest in 2014 (see Table 2).

**Table 2:** Trend in the proportions of influenza positive tests by clinical and demographic characteristics and by year.

	2014		2015		2016		Yearly growth ratio†	p-value
Characteristic	SARI	Flu positive	SARI	Flu positive	SARI	Flu positive		
	N	N (%)	N	N (%)	N	N (%)		
Total	168	7 (4.2)	1132	44 (3.9)	697	26 (3.7)	0.95 (0.66–1.37)	0.787
Age in months*								0.054
< 6	29	0 (0.0)	246	6 (2.4)	160	3 (1.9)	1.10 (0.36–3.33)	
6–11	20	0 (0.0)	184	7 (3.8)	127	7 (5.5)	1.72 (0.68–4.34)	
12–23	41	1 (2.4)	245	14 (5.7)	158	6 (3.8)	0.94 (0.48–1.84)	
24–59	49	1 (2.0)	291	10 (3.4)	153	8 (5.2)	1.56 (0.73–3.29)	
5Yr - 14Yr	29	5 (17.2)	166	7 (4.2)	99	2 (2.0)	0.31 (0.14–0.69)	
Gender**								0.903
Male	92	5 (5.4)	591	24 (4.1)	393	15 (3.8)	0.87 (0.54–1.39)	
Female	71	2 (2.8)	492	18 (3.7)	279	10 (3.6)	1.06 (0.58–1.91)	
No information	5	0 (0.0)	49	2 (4.1)	25	1 (4.0)	-	
**Season of case detection**								< 0.003
Wet	63	6 (9.5)	463	25 (5.4)	267	6 (2.2)	0.49 (0.29–0.82)	
Dry	105	1 (1.0)	669	19 (2.8)	430	20 (4.7)	1.79 (1.05–3.05)	
**Signs at hospitalization**								
Self-reported fever	110	6 (5.5)	393	18 (4.6)	397	21 (5.3)	1.46 (0.67–3.19)	0.582
Difficult breathing	128	2 (1.6)	691	31 (4.5)	331	11 (3.3)	1.38 (0.66–2.91)	0.662
Measured fever (> 38C)	5	0 (0.0)	45	3 (6.7)	25	2 (8.0)	1.62 (0.35–7.63)	0.799
**Symptoms at hospitalization**								
Cough	145	7 (4.8)	994	43 (4.3)	669	26 (3.9)	0.90 (0.02–43.67)	0.841
Sore throat	15	0 (0.0)	80	4 (5.0)	6	0 (0.0)	1.65 (0.18–15.23)	0.874
Runny nose	66	3 (4.5)	641	27 (4.2)	397	17 (4.3)	0.14 (0.55–2.38)	0.889
Hospitalization motive**								
Bronchopneumonia	102	1 (1.0)	644	27 (4.2)	461	19 (4.1)	2.17 (1.01–4.65)	0.129
Pneumonia	30	1 (3.3)	143	1 (0.7)	78	2 (2.6)	1.26 (0.25–6.30)	0.899
Bronchitis	21	0 (0.0)	145	2 (1.4)	93	2 (2.2)	2.07 (0.35–12.18)	0.695
Other***	33	5 (15.2)	251	14 (5.6)	99	4 (4.0)	0.40 (0.17–0.90)	0.087
Previous or currently diagnosed asthma	32	1 (3.1)	339	19 (5.6)	187	3 (1.6)	0.38 (0.16–0.89)	0.074
**Treatments**								
Antibiotics	50	2 (4.0)	728	30 (4.1)	666	25 (3.8)	0.93 (0.59–1.46)	0.880
Oxygenation	2	0 (0.0)	134	6 (4.5)	103	6 (5.8)	1.51 (0.48–4.75)	0.730
Other (no oxygenation neither antibiotics)****	117	5 (4.3)	380	11 (2.9)	29	1 (3.4)	0.86 (0.31–2.37)	0.767
**Outcome**								
Death	0	-	5	1 (20.0)	0	-	-	-
Recovered	168	7 (4.2)	1127	43 (3.8)	697	24 (3.4)	0.96 (0.67–1.38)	0.818

*M—months; Yr—years

** Some cases had multiple diagnoses

*** Malaria, Oral Candidiasis, Anemia, Acute Gastroenteritis, Marasmus, Kwashiorkor, Malnutrition and Congenital Cardiopathy

**** Mechanical ventilation and admission to the Intensive Care Unit.

† Yearly growth ratio represents the yearly relative average trend of the proportion of influenza positivity. If > 1 is an increasing, if < 1 is a decreasing trend. The trend is estimated from log-binomial regression with calendar time, the dummy of the characteristic and interaction of calendar time and the dummy indicators. The exponentiated linear combination of the time coefficient and the interaction is the yearly increase. The p-values are the overall significance of the interaction.

### Trends and seasonality of influenza between 2014 and 2016

Of the 77 SARI patients with confirmed influenza, 63.6% (49/77) had their CRF completely filled out.

**Fig 3:**
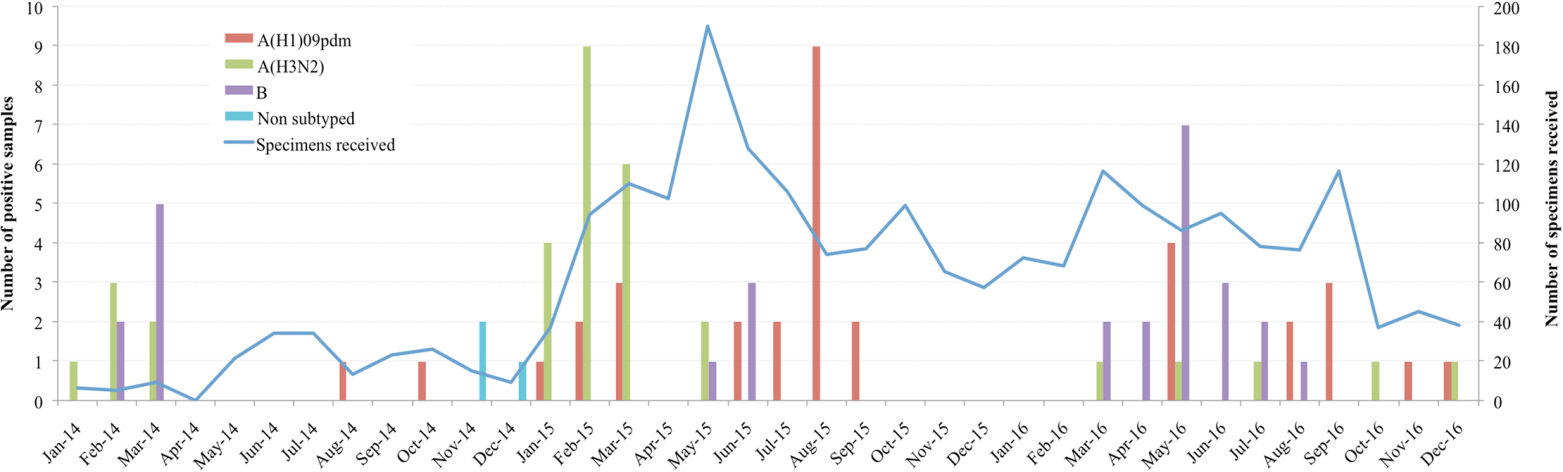
Monthly variation of influenza virus types and subtypes and positivity rates from January 2014 to December 2016. Nasopharyngeal and oropharyngeal swabs from children admitted with SARI were tested for Influenza virus using RT-PCR.

Data from Table 2 shows that although the number of children with SARI who were enrolled in 2014, 2015 and 2016 was heterogeneous, the percentage influenza-positive was similar across the different years [the percentage influenza-positive in 2014, 2015 and 2016 was 4.2% (7/168), 3.9% (44/1132) and 3.7% (26/697), respectively]. Notably, from January 2014 through December 2016 there was a substantial monthly variation in the frequency of influenza virus, reaching up to 60% - 80% during the peaking of cases of influenza (Fig 3).

Fig 3 also shows that there were seasonal variations in the frequency of influenza virus. The curve of temporal distribution of cases of influenza had a bimodal shape, with two peaks each year, one peak in the rainy season and second peak in the dry season, but the magnitude of each peak was heterogeneous in different years.

No significant difference was observed in the distribution of cases of influenza between 2014 and 2016 for the following variables: age, gender, the season of detection, signs, and symptoms, clinical diagnostic at admission and medical procedure. However, across different years there was a trend towards the higher frequency of influenza in males as compared to female.

Of the 5 deaths among children with SARI, 1.3% (1/78) and 0.3% (4/1913) occurred in influenza-positive and negative children, respectively (see Table 1) and 40% (2/5) were sons of HIV positive mothers (See S2 File).

### Influenza type and sub-types

Among all influenza positive cases, 33.7% (26/77), 35.1% (27/77) and 28.6% (22/63) were caused by influenza A/H3N2, A/H1N1pdm09, and B, respectively (S2 File). All subtypes circulated in 2014, 2015 and 2016 (Fig 3), however in 2014 the dominant subtypes were H3 and B, in 2015 were H1 and H3, while in 2016 were H1 and B.

## Discussion

Despite the great effort and progress that has been made in recent years to understand the epidemiology of influenza in sub-Saharan Africa, the disease still remains poorly understood in many countries, including Mozambique. In this study we found a frequency of influenza of 3.6% in children admitted to pediatric wards in two hospitals in Maputo city, which is consistent with reports from few other studies conducted in sub-Saharan Africa [26, 27], but lower than that reported in most of studies conducted in the region [10, 28], reinforcing that epidemiology of influenza is highly heterogenetic in different countries.

Notably, the frequency of influenza found in our study was also lower than that reported in two other studies conducted in Manhiça District Hospital, situated in a rural district in southern Mozambique, which found a prevalence of influenza of 15% and 8% in Feb1999-May2000 and Sep2006-Sep2007, respectively [20, 21]. On the other hand, the study conducted by O'Callaghan-Gordo et al enrolled only outpatient children [21], while our enrolled children with severe illness, which can also partially explain the difference because several studies in other countries in the region have shown consistently that prevalence of influenza is higher among outpatient children (non severe) as compared to hospitalized (severe) [11, 29–31].

Regarding the age, the frequency of influenza positive cases was higher in children less than 5 years of age, which is similar to findings reported in other studies[13, 32, 33].

We also compared study groups in terms of signs and symptoms, and data from this study showed that a cough, self-reported fever, measured fever (≥38^°^C) and difficulty breathing were associated to influenza positivity, corroborating findings from previous studies in other countries [25, 34, 35].

In terms of seasonality of influenza, our data demonstrated that influenza virus occurred throughout the year in Mozambique, and presented a bimodal curve shape with two peaks, one in the dry season and another in the rainy season, being consistent with data from other sub-Saharan Africa countries[10, 16, 18, 36, 37]. This highlights that in Mozambique all effort to prevent influenza, such as vaccination should be considered throughout the year as suggested by others [12, 37].

Deaths occurred in children younger than 5 years old with bronchopneumonia, highlighting that particular attention should be paid to these children. Other authors had shown similar findings [27, 38].

During the revision of clinical files of deceased children we found that HIV results of the children were not available, but two of them were born from HIV seropositive mother, raising serious concern, because the prevalence of HIV in Mozambique is very high (13,5%) [39] and 100,000 children are estimated to be infected with HIV [40, 41]. Indeed, recent studies from South Africa and Malawi, which are close to Mozambique and where the prevalence of HIV is also very high, showed that HIV was significantly associated with progression to severe respiratory illness [32, 42, 43]. However, we acknowledge that this study was not designed to assess the impact of HIV on influenza and for this reason, we recommend that studies to assess this aspect should be urgently conducted in Mozambique.

In this study, we also noted that no difference was found in the frequency of asthma among influenza positive and negative children. However, previous studies have shown a relationship between influenza and asthma exacerbation [44].

Our study is the first to assess the distribution of types and sub-types of influenza circulating in children with SARI in Mozambique and showed that both the seasonal (A/H3N2) and the seasonal (A/H1N1pdm09) are prevalent in Mozambique, but the dominant influenza strain varied in different years. A similar pattern was also seen in other studies conducted in the region [36].

We would like to acknowledge few limitations of our study, such as the fact that not all eligible children who attended these two hospitals during the recruitment period were enrolled, however, to minimize selection bias, each day we recruited the first three eligible children attending these hospitals. Information on the presence of concomitant bacterial infections was not available, and for this reason, is difficult to address properly the high level of consumption of antibiotic noted in this study. Lastly, information about HIV status of each child’s mother was not available for all participants.

## Conclusion

Taking together, our results show that influenza virus is prevalent in children with SARI who lives in a large urban/sub urban area in Mozambique, despite that its frequency was lower than that reported in most of the studies conducted in the region. Cases of influenza occurred throughout the year with a bi-modal curve shape and the fatality occurred in children younger than 5 years old. Results of this study will drive national efforts to prevent and improve care to influenza-infected children, such as training of clinicians in order to improve their knowledge on diagnosis and clinical management of potential cases of influenza, vaccination against influenza and use of antivirals in high-risk groups. Expansion of routine SARI surveillance to other regions of the country is also needed for better understanding of geographical differences.

## Supporting information

S1 File Case investigation form. (TIF)

S2 File Minimal data set. https://dataverse.harvard.edu/privateurl.xhtml?token=20c1b910-9af3-4cfa-97ad-9b2efa22c466.
(ZIP)
