# Supplementary figures and images for: Clinical and epidemiological characterization of influenza virus infections in children with severe acute respiratory infection in Maputo, Mozambique: Results from the implementation of sentinel surveillance, 2014 – 2016
Source: PLoS One. 2018 Mar 28;13(3):e0194138. doi: 10.1371/journal.pone.0194138 (PMC5874022; doi:10.1371/journal.pone.0194138)

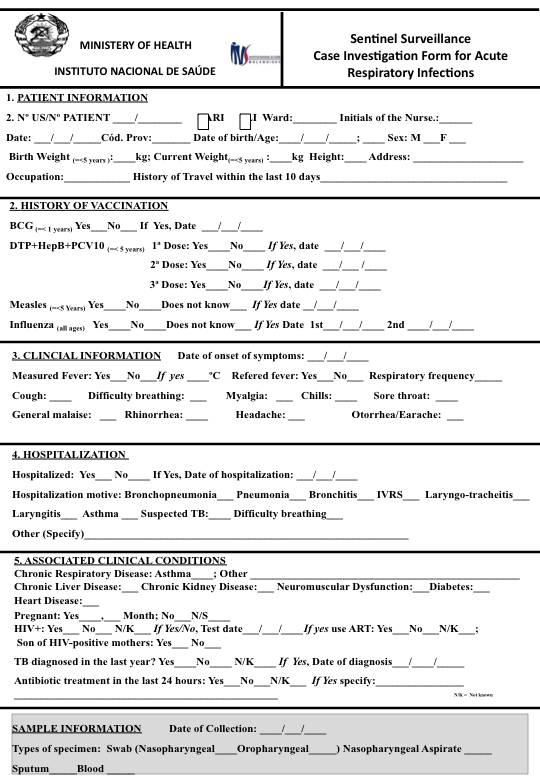

Supplement: S1 File — (TIF) [file pone.0194138.s001.tif]
